# Decreased Expression of LAMB3 Is Associated with Esophageal Cancer Stem Cell Formation

**DOI:** 10.34172/apb.2022.084

**Published:** 2021-09-29

**Authors:** Anoosheh Ehtesham, Ayyoob Khosravi, Marie Saghaeian Jazi, Jahanbakhsh Asadi, Seyyed Mehdi Jafari

**Affiliations:** ^1^Metabolic Disorders Research Center, Golestan University of Medical Sciences, Gorgan, Iran.; ^2^Stem Cell Research Center, Golestan University of Medical Sciences, Gorgan, Iran.; ^3^Department of Molecular Medicine, Faculty of Advanced Medical Technologies, Golestan University of Medical Sciences, Gorgan, Iran.; ^4^Department of Biochemistry and Biophysics, School of Medicine, Golestan University of Medical Sciences, Gorgan, Iran.

**Keywords:** Esophagus cancer, Cancer stem cell, LAMB3 protein, Topoisomerase II alpha

## Abstract

***Purpose:*** Esophageal squamous cell carcinoma (ESCC) is a highly aggressive cancer. The main cause of death in ESCC is related to relapse, metastasis, and resistance to cancer therapy. Recent studies have shown that a minor subset of cancer cells, known as cancer stem cells (CSCs), are responsible for tumor formation initiation and cancer progression. Understanding the genes associated with CSCs and metastasis can help in targeted cancer therapy. The aim of this study was to assess the expression of LAMB3 and TOP2A metastasis-associated genes in CSCs and adherent cells in the xenograft mouse model.

***Methods:*** Esophageal CSCs were enriched by the sphere formation method. The expression level of LAMB3 and TOP2A genes were evaluated in spheres and adherent cells *in vitro* by qRT-PCR. A xenograft mouse model was established to investigate the tumorigenesis and metastasis potential by subcutaneous and tail vein injection of CSCs and adherent YM-1 cells. Consequently, LAMB3 and TOP2A expression at the mRNA level was assessed in tumors. Immunohistochemistry was also used to evaluate the LAMB3 expression at the protein level in tumors.

***Results:*** CSCs-derived tumor was developed more quickly than the adherent cells-derived tumor. LAMB3 at mRNA and protein level was significantly down-regulated in sphere-derived tumor compared with adherent cells-derived tumor (*P* value <0.05). TOP2A expression was almost similar in both sphere cells and adherent cells and there was no significant difference.

***Conclusion:*** we concluded that YM-1 spheres have CSCs characteristics *in vitro* with high capability of tumorigenicity in vivo. Our results were also shown that the LAMB3 expression was decreased in YM-1 spheres suggesting LAMB3 association with sphere formation.

## Introduction

Esophageal cancer (EC) is the eighth among the most common malignancies and the sixth cause of death from cancer worldwide.^1^ Esophageal squamous cell carcinoma (ESCC), a major subtype of EC, is considered as highly aggressive malignancy of the digestive tract.^2^ Despite recent advances in radiotherapy, chemotherapy and surgical therapeutic approaches, the 5-year survival rate of ESCC patients is still less than 15%.^3^

Recently, studies have shown that a limited subset of cancer cells, known as cancer stem cells (CSCs) or tumor-initiating cells play a critical role in tumorigenicity and metastasis.^4^ The CSCs, similar to normal stem cells, are able to self-renew and have differentiation potential to different cell lineages.^4^ The CSCs take part important function in tumorigenesis and even low numbers of them are able to initiate primary tumor formation and metastasis progression.^5^ These small subpopulation have been already identified in many solid tumors^4^ including EC.^6^ Considerably, Gene expression profile in CSCs is exclusive and necessary to initiate and progress tumors and metastasis.^7^

Different metastasis-associated genes are playing important roles in high invasive ability of CSCs. LAMB3 encodes β3, one of the three chains of Laminin-332, which have various functions in invasion, migration and progression of metastasis in different cancers.^8^ The MMP-7 can produce a cleaved fragment of 90 kD originated from LAMB3 which can induce the migration of colon carcinoma cells.^9^ Moreover, during the pulmonary metastasis early arrest of tumor cells happens through the interaction of α3β1 integrin with LN-332.^10^

The second gene in our study is TOP2A that encodes 170kD topoisomerase IIa (topoIIa) which is involved in DNA synthesis, gene transcription and DNA topology.^11,12^ It plays a critical role in development, proliferation, and invasion in carcinomas.^13,14^ Interestingly, dysregulation of TOP2A is significantly associated with tumor proliferation and progression such as, pancreatic^15^ and EC.^16^

Recent studies have more evaluated LAMB3 and TOP2A expression in malignant tumors rather than in CSCS. Studies have shown that LAMB3 and TOP2A were overexpressed in malignant tissues compared with normal tissues in ESCC.^17^ In the current study, we aimed to investigate the expression of these two metastasis-associated genes in CSCs of ESCC *in vitro* and *in vivo*.

## Materials and Methods

###  Cell culture

Human ESCC YM-1 cell line has already been established in our laboratory at Golestan University of Medical Sciences.^18^ Cells were cultured as a monolayer in high glucose DMEM/F12 (BioIdea, CN:1027) supplemented with 10% fetal bovine serum (FBS) (BioIdea, CN:10270-106), 100 IU/mL penicillin and 100 μg/mL streptomycin (BioIdea, CN:1036) at 37°C in a humidified 5% CO2 incubator. The medium was changed every 3 days and cells were passaged to another flask every week.

###  Sphere-forming assay

 To obtain sphere cultures, monolayer cells in DMEM/F12 medium containing FBS 5% were washed with PBS (BioIdea). The cells were dissociated using Trypsin-EDTA 0.25% (BioIdea, CN:1002). Subsequently, the dissociated cells were seeded at 20,000 cells/mL in the low attachment petri dish (Labtrone, Iran). In petri dishes, the cells were suspended in serum-free DMEM/F12 supplemented with antibiotics, 20 ng/mL of human epidermal growth factor (EGF; ROYAN, CN:1102-25), 20 ng/mL of human basic fibroblast growth factor (bFGF; ROYAN, CN:1101-25), and 2% B-27 50x supplement (GIBCO, CN:17504-044).

###  Sphere passaging

 Spheres were passaged up to five times every 3-4 days. The process of sphere passaging takes approximately 15 to 20 days. The spheres were collected from petri dish gently, then dissociated with trypsin-EDTA. The single cells were then centrifuged and re-suspended in a new petri dish containing serum-free medium and growth factors. The number and volume of spheres were counted by Trypan blue staining under an optical microscope.

###  Subcutaneous xenograft mouse model

YM-1 parental and sphere-forming cells were divided into two groups. The number of 10^6^ single cells in PBS 100 μL/Matrigel 100 μL (1:1) mixture were subcutaneously injected into 4week-old male athymic nude mice weighing 16-18 grams. The nude mice were maintained under specific pathogen-free conditions in Pasteur Institute, Amol, Iran. When the tumor size achieved to minimum 1 cm in diameter, mice were sacrificed. The primary tumors were harvested and minced into small pieces. Part of the tissue was used for the molecular experiment and others were fixed in 10% formalin then embedded in paraffin for histological and immunohistological staining. To ensure the tumor pathology, the H&E staining was performed in the 5 Azar hospital laboratory, Gorgan, Iran.

###  Experimental metastatic xenograft mouse model

To carry out the experimental metastatic mouse model, cells were injected into the nude mice via the lateral tail vein and intraperitoneal. Sphere cells in PBS (10^6^ cells per mouse) and adherent cells in PBS (10^6^, 3×10^6^ and 5×10^6^ cells per mouse) were used in separate groups. Moreover, for more assurance, intraperitoneal injection also was checked for metastasis experiments (10^6^ cells per mouse). Animals were euthanized when the moribund signs were observed in them or after 3 months of injection if they survived. Lungs, liver, femur, and bone were isolated and fixed in 10% formalin for hematoxylin and eosin (H&E) staining. The stained sections were observed under the microscope.

###  RNA extraction, reverse transcription and quantitative real-time PCR ( qRT -PCR) 

 Total RNA was isolated from cells and tumors using TRIZOL (Invitrogen CN:15596-026). The first strand cDNA was synthesized using a reverse transcriptase kit (Yekta tajhiz) following the manufacturer’s protocol. Then qPCR was performed using SYBR Green PCR Master Mix kit (Applied Biosystems, CN:4367659) by ABI 7300 Real time PCR instrument. The GAPDH gene was used as housekeeping control to normalize gene expression level. GAPDH, LAMB3 and TOP2A were amplified using the following thermal condition: 95°C for 5 minutes, 38 cycles of 95°C for 10 seconds, 60°C for 30 seconds and 72°C for 40 seconds following with melt curve analysis and thermal condition for SOX2 and NANOG was 95°C for 2 minutes, 38 cycles of 95°C for 15 seconds, 62°C for 1 minutes and 72°C for 1.30 minutes and final extension 72°C for 10 minutes. Expression of genes was measured using 2−ΔCt. Experiments were performed in triplicate.

 The sequence of used specific primers were as following: LAMB3 forward: 5’-GTA TGG CGA GTG GCA GAT GA-3’ and reverse: 5’-GCA GAG AGA CAG GGT TCA CA-3’, TOP2A forward: 5’-GCA TTC CTA CAT CCA AGG GTG G-3’ and reverse: 5’-TGT CTG AGA GTC AAA GGT TGG GTT-3’, GAPDH forward: 5’-AAG CTC ATT TCC TGG TAT GAC AAC-3’and reverse: 5’-CTC TCT TCC TCT TGT GCT CTT G -3’, SOX2 forward: TACAGCATGTCCTACTCGCAG and reverse: GAGGAAGAGGTAACCACAGGG and NANOG forward: ATTCAGGACAGCCCTGATTCTTC and reverse: TTTTTGCGACACTTCTCTGC

###  Immunohistochemistry (IHC) 

 To assess the LAMB3 protein expression at tissue sections, the fixed xenograft tumor sections from adherent and sphere cells were stained using the anti-LABM3 antibody. Formalin-fixed and paraffin-embedded tumor sections with 5 µm thickness were used for staining. The sections were deparaffinized with xylene and then were rehydrated with ethanol. Antigen retrieval was used to break the possible methylene bridges. The sections were washed with buffer and then were incubated with 3% H2O2 and 1% bovine serum albumin (BSA) respectively. Slides were then placed in a humid chamber. Slides were stained with the primary mouse monoclonal antibody against LAMB3 (Santa Cruz, sc-133178) diluted 1:50 for overnight. Subsequently, slides were washed with buffer and incubated with a mouse IgGk protein conjugated to horseradish peroxidase (m- IgGk-HRP, sc-516102). The stained xenograft tumors tissue sections were then incubated with DAB solution. Finally, the slides were counterstained by hematoxylin and were studied using light microscopy.

###  Statistical analysis

Two-tail Student’s *t* test and ANOVA were used to analyze differences between groups. In the case of non-normal distribution, the non-parametric test was used. All statistical analysis was performed by SPSS 16.0 software. P-values of less than 0.05 were recognized as statistically significant. The graphs were drawn by GraphPad Prism v 5.04.

## Results and Discussion

###  The enriched YM-1 spheres show CSCs characteristics 

CSCs are tumor initiating-cells that are able to generate heterogeneous cell population within a tumor.^19^ In a cancer cell line, CSCs can be identified with different features including sphere formation assay, over-expression of stemness genes, increased drug resistance and high *in vivo* tumorigenesis potential.^20,21^ In sphere forming-assay, CSCs subpopulation can be enriched and have self-renewal and independent growth *in vitro*.^21,22^

**Figure 1:**
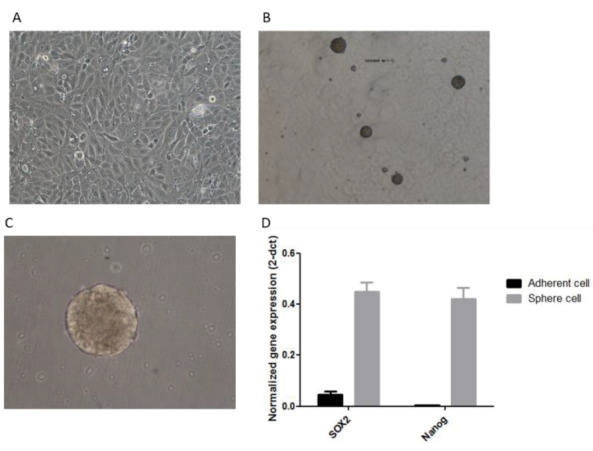
YM-1 cells in special conditions represent features of CSCs.(A) adherent cells in DMEM/F12 medium supplemented with 10% FBS. (B and C) Sphere clusters in specific 3D culture (×4 and ×40). (D) mRNA level of SOX2 and NANOG are up-regulated in spheres vs adherent cells.Bars represent the normalized gene expression (2^-dct^) with standard error bars

In this study, to evaluate the presence of CSCs, we isolated CSCs from YM-1 EC cells with sphere formation assay. YM-1 adherent cells were cultured in serum-free, low attachment three-dimensional (3D) culture medium. Subsequently only a limited number of cells were able to grow and self-renew (Figure 1A). In this condition, single cells formed sphere clusters as shown in (Figure 1B and 1C).

The spheres were checked for stemness gene expression and as shown in (Figure 1D), significant overexpression of the SOX2 and Nanog was observed in YM-1 sphere cells. The sphere formation capability of the YM-1 cells was shown previously in different projects in our lab but checked again in the current study.

###  Sphere-forming cells exhibit higher tumorigenicity compared with parental cells in vivo

**Figure 2:**
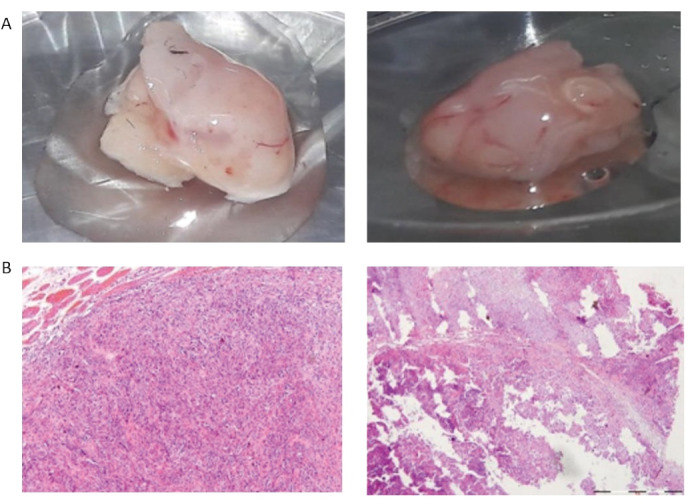
The high tumorgenicity ability of spheres. (A) The spheres-derived tumor after only a month reached the same tumor size (1 cm in diameter) of adherent cells-derived tumor following 2 months. (B) The H&E staining of xenograft tumors confirmed the neoplastic histology of the tumors (×4)

For more confirmation that the YM-1 spheres have CSCs features, we aimed to develop xenograft mouse model with subcutaneous injection. The results indicated 10^6^ enriched YM-1 spheres formed a tumor measuring 1 cm in diameter after one month while 10^6^ adherent cells formed a tumor in this size after two months (Figure 2A). The histology of both tumors was confirmed as ESCC tumor by (H&E) staining (Figure 2B). At the end, our findings confirmed that the spheres subpopulation of the YM-1 EC cells harbors most of the characteristics of CSCs.

###  YM-1 cell line metastatic potential

To assess the metastatic potential of ESCC YM-1cell line, we developed a xenograft mouse model by experimental metastatic assay in which cells were injected directly into the systemic circulation.^23^ Mice were injected with YM-1 sphere or adherent cells via a tail vein and intraperitoneal injection. Then they were sacrificed following maximum 3 months follow up and all of the organs were isolated. Since no nodule was found we stained lungs, liver, femur, and bone of the mice with H&E staining to make a more accurate assessment. There was no indication of metastasis under the microscope (data not shown). Due to our results, we conclude that probably YM-1 cell line or spheres have poor ability to form secondary metastatic tumors which could be explained with the cell-specific characteristics.

###  LAMB3 expression assessment at mRNA level in spheres and adherent cells 

Interaction of CSCs with their microenvironment can lead to cancer development and metastasis. extracellular matrix (ECM) is a major structure of CSCs niche that its protein components could be dysregulated in pathological states and cancer.^24^ LAMB3 by coding β3 is involved in production of Laminin-332 as a ECM protein. Laminin-332 is made up of three subunits (α3, β3 and γ2). Studies have shown each of Laminin-332 subunits lonely, either as a dimer or as a trimer plays role in stimulating invasion, migration and homing in metastasis of colon,^9^ pulmonary cancers^10^ and esophagus squamous cell carcinoma.^25^ However, different studies reported either up-regulation or down-regulation of Laminin-332 heterotrimer in different cancers.^26^

**Figure 3:**
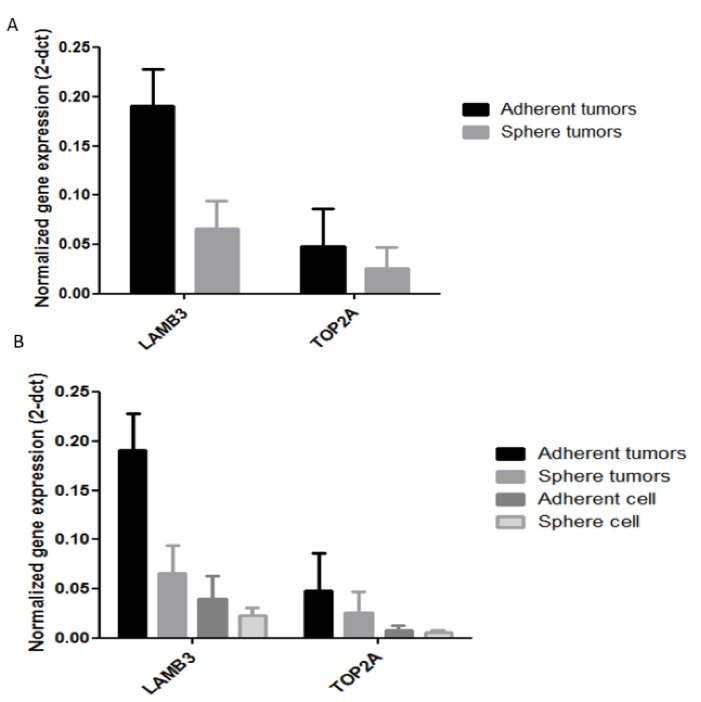
Down-regulation of LAMB3 and TOP2A in spheres vs adherent cells.(A and B) qRT-PCR analysis indicated Down-regulation of LAMB3 and TOP2A in spheres compared with adherent cells in vivo and in vitro respectively

Upregulation of LAMB3 is correlated with the depth invasion and metastasis risk of some malignant tumors such as the colorectal^27^ and gastric.^8^ Up to now, limited studies have examined the expression of LAMB3 and Laminin-332 in CSCs while in current study, we compared the expression of LAMB3 in YM-1spheres with CSCs features withadherent cells* in vitro* and *in vivo* by qRT-PCR method. As shown in (Figure 3B) the gene expression of LAMB3 was decreased in spheres compared with adherent cells *in vitro*, however, it was not significant (*P* value > 0.05). To investigate LAMB3 expression *in vivo*, we evaluated the expression at mRNA level in spheres-derived tumor and adherent cells-derived tumor in subcutaneous injection (primary tumor). Similar to *in vitro* results, LAMB3 expression in the spheres-derived tumor was decreased significantly (*P* value = 0.03) (Figure 3).

###  Xenograft tumor derived from spheres express less LAMB3 proteins

**Figure 4:**
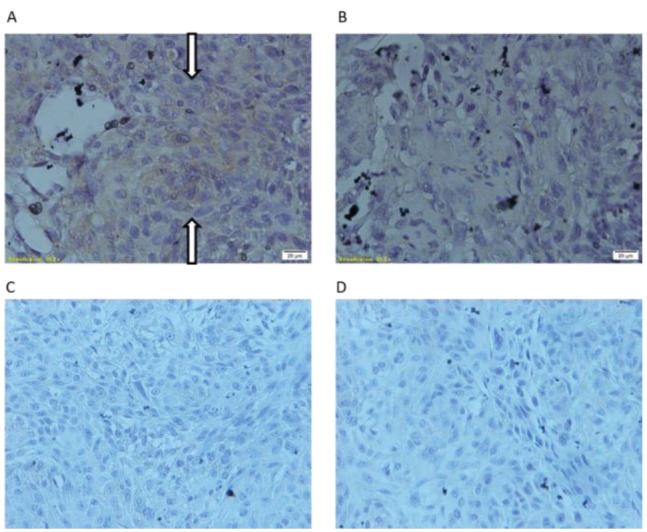
Comparison of the protein expression level of LAMB3 in spheres and adherent cells *in vivo*.(A) Immunohistochemistry (IHC) analysis demonstrated significant down-regulation of LAMB3 in spheres-derived tumor vs adherent cells-derived tumor. (B) Up-regulation of LAMB3 expression at the protein level in adherent cells-derived tumor is showed with flash. (C and D) negative control samples

Whereas there was a meaningful difference in LAMB3 mRNA expression between two tumors, we evaluated LAMB3 expression in protein level in two different tumors by IHC. Our observations represented that LAMB3 protein is detectable in both spheres-derived tumor and adherent cells-derived tumor. The spheres-derived tumor expresses less LAMB3 protein level in comparison to the adherent cells-derived tumor (Figure 4), which is fallowing the qRT-PCR results.

Oktem et al^28^ reported that LAMB3 expression is significantly upregulated in CD133+/CD44+CSCs grown as a monolayer comparing with CD133-/CD44-counterpart. Govaere et al^29^ showed the role of Laminin-332 in sustaining CSCs quiescence in hepatic carcinoma. Liu et al^30^ indicated that Laminin-332 promotes tumor metastasis and have a prominent role in the progression of lung cancer. In the study of Liu et al,^30^ Laminin-332 was up-regulated in tumor spheres compared to adherent cells, consequently, only tumor spheres resulted in lung metastasis via tail vein injection. However, our data showed that the tumors derived from sphere cells with higher tumorigenic features express less LAMB3, both at mRNA and protein level that this significant down-regulation in expression shows relation and influence of LAMB3 insphere formation andEC progression. concordantly, Lathia et al^31^ showed that LAMA2 expression is downregulated in CSC of glioblastoma and LAMA2 is identified as a malignant marker in aggressive ependymoma.

###  TOP2A expression assessment at mRNA level in spheres and adherent cells 

Another gene studied in current study was TOP2A, the alpha subunit of the topoisomerase II which is the target of cancer therapy drugs such as anthracyclines.^32^ Inhibition of TOP2A suppresses metastatic spread of tumors.^15^ TOP2A expression have been rarely investigated in CSCs or tumor spheres and more commonly evaluated in cancer tumors in which TOP2A expression was correlated with advanced stages of the disease.^33^ In this study, we also evaluated TOP2A expression at mRNA level in spheres and adherent cells *in vitro* and in subcutaneous tumors. Our findings showed non-significant downregulation of the TOP2A in spheres of both *in vitro* and *in vivo* experiments compared with adherent cells (Figure 3).However, previous studies reported TOP2A association with tumor proliferation and progression in several types of cancers such as pancreatic,^15^ prostate,^33^ and ESCC.^16^ Terashima et al^34^ indicated that TOP2A expression markedly increases the risk of hematogenous recurrence and peritoneum recurrences in gastric carcinoma. Li et al^33^ illustrated that TOP2A^high^ cells related to recurrence/ metastasis but unexpectedly TOP2A^neg^ cells show CSCs properties in prostate cancer which is in concordant to the decreased expression of TOP2A in esophageal CSCs observed in our findings.

## Conclusion

In conclusion, our results illustrated that YM-1 esophageal cancer cell spheres have CSCs characteristics *in vitro* with high capability of tumorigenicity in vivo. We found that the LAMB3 gene and protein expression is decreased in YM-1 spheres suggesting LAMB3 association with sphere formation and esophageal CSC development; however the underlying mechanism remains to be investigated in future experiments.

## Acknowledgments

 This study was supported by the Metabolic Disorders Research Centre, Golestan University of Medical Sciences (No.960413075).

## Ethical Issues

 In this project, nude mice were kept and treated at Pasteur Amol institute according to health instructions and it was approved in local ethic committee (No: ir.goum.rec.1395.276).

## Conflict of Interest

 All authors declare no conflicts of interest in this work.
